# Evaluating the effectiveness of prompt engineering for knowledge graph question answering

**DOI:** 10.3389/frai.2024.1454258

**Published:** 2025-01-13

**Authors:** Catherine Kosten, Farhad Nooralahzadeh, Kurt Stockinger

**Affiliations:** School of Engineering, Institute of Computer Science, Intelligent Information Systems Research Group, Zurich University of Applied Sciences, Winterthur, Switzerland

**Keywords:** knowledge graph question answering, SPARQL, RDF, LLMs, prompt engineering

## Abstract

Many different methods for prompting large language models have been developed since the emergence of OpenAI's ChatGPT in November 2022. In this work, we evaluate six different few-shot prompting methods. The first set of experiments evaluates three frameworks that focus on the quantity or type of shots in a prompt: a baseline method with a simple prompt and a small number of shots, random few-shot prompting with 10, 20, and 30 shots, and similarity-based few-shot prompting. The second set of experiments target optimizing the prompt or enhancing shots through Large Language Model (LLM)-generated explanations, using three prompting frameworks: Explain then Translate, Question Decomposition Meaning Representation, and Optimization by Prompting. We evaluate these six prompting methods on the newly created Spider4SPARQL benchmark, as it is the most complex SPARQL-based Knowledge Graph Question Answering (KGQA) benchmark to date. Across the various prompting frameworks used, the commercial model is unable to achieve a score over 51%, indicating that KGQA, especially for complex queries, with multiple hops, set operations and filters remains a challenging task for LLMs. Our experiments find that the most successful prompting framework for KGQA is a simple prompt combined with an ontology and five random shots.

## 1 Introduction

Since the release of ChatGPT in November of 2022, Large Language Models (LLMs) have seen a surge in popularity. One of the novel aspects of this, is that unlike previous iterations of “chatbot-like” technology, ChatGPT has actually seen sustained interest and use from users in all aspects of life.^1^ The popularity of ChatGPT for question answering underscores the research value of chat-based interfaces, demonstrating real-world user demand and justifying continued research efforts especially for Knowledge Graph Question Answering (KGQA).

Large Language Models like ChatGPT can already perform a multitude of tasks in domains from business to education (Bahrini et al., 2023). However, one flaw in LLMs that has garnered attention in the last year is that they hallucinate, meaning LLMs do not always provide factual answers. While accuracy is not essential to some tasks, such as creative ideation and some writing or administrative tasks, accuracy is of utmost importance when using an LLM in factual question answering tasks such as question answering against databases or knowledge graphs (Zhang et al., 2023; Nooralahzadeh et al., 2024; Fürst et al., 2024).

**Figure 1:**
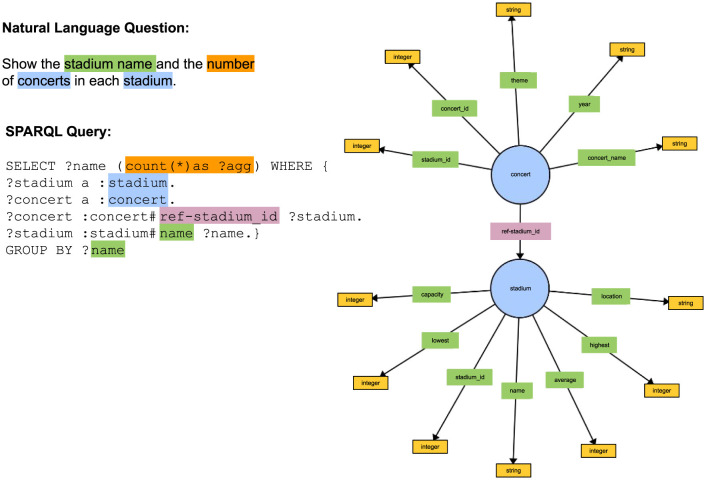
This figure shows an example of a natural language question, its corresponding SPARQL query and a section of a knowledge graph with the classes and properties used in the SPARQL query.

Assume that we have a knowledge graph (KG) about concerts and stadiums as shown in Figure 1. Further, assume that we want to answer the natural language question *Show the stadium name and the number of concerts in each stadium*. The major task of KGQA is to translate the natural language question into the formal query language that the KG supports, such as SPARQL.

LLMs being used as KGQA systems tend to hallucinate incorrect class names, property names and literals in the generated SPARQL queries. Some class and property names as well as literals may also be completely made up by the LLM, in which case, the knowledge graph would not return an answer to the user (Kosten et al., 2023). Hallucinations can also include class and property names as well as literals that exist in the graph but do not match the question being asked and would return an incorrect answer. In this paper, specifically target mitigating both forms of hallucination by using various approaches of prompt engineering to improve the performance of LLMs as KGQA systems.

An interesting byproduct of the LLM boom is that many researchers in the field have begun to see training data as obsolete, due to the fact that LLMs are trained on Terabytes of data. The reasoning behind this is, if they have been trained on Terabytes of data what else could we possibly train them on? We show that for the task of KGQA, training data is still highly relevant and significantly improves few-shot results from 8% to 51% for LLMs compared to a zero-shot baseline.

Overall, this paper makes the following contributions:

- We design and explore the effects of various prompt engineering approaches on the task of Knowledge Graph Question Answering for a complex data set.
- We assess the prompting methods using the Spider4SPARQL benchmark (Kosten et al., 2023) and compare GPT-3.5 and Code Llama, revealing that even the best models struggle to surpass 51% accuracy on complex queries.
- We identify the most effective prompting framework for the KGQA task – a simple prompt combined with an ontology and five random shots – and conduct thorough error analysis to pinpoint areas for improving KGQA.

## 2 Knowledge graph question answering

Despite decades of research, KGQA is still a significant challenge for the scientific community. Systems that can perform KGQA have evolved over the years from earlier rule-based approaches (Sima et al., 2021) to traditional machine learning methods with ensemble techniques (Singh et al., 2018) and most recently deep learning. Early deep learning KGQA systems employed architectures like Tree-LSTMs (Liang et al., 2021). Modern KGQA systems use the most recent deep learning architectures like the cutting-edge transformer as seen in Large Language Models.

There are a few main components necessary to build and use a KGQA system. The first element is the knowledge graph (KG) itself, which consists of an ontology and underlying data (literals), also known as the Terminological Box (T-box) and the Assertion Box (A-box), respectively (Krotzsch et al., 2014). The next component is a dataset that can be used for training and evaluating a KGQA system. This resource contains natural language questions with their corresponding SPARQL queries, serving as a benchmark for assessing the systems' performance. The last building block is the KGQA system which translates a natural language question into a SPARQL query.

### 2.1 Translating a natural language question into SPARQL

The first step in the task of translating a natural language question into SPARQL is mapping the entities in the natural language question to the classes, properties, and literals in the knowledge graph. As Figure 1 shows, the system must be able to recognize that *stadium name* from the natural language question refers to the Datatype property *name* in the *stadium* Class. It also needs to find the Class *concert* and be able to connect it to the Class *stadium* with the correct Object Property, in the correct direction, as shown in Example 1.

**Example 1:**

$\begin{array}{c} concert \end{array}\to \begin{array}{c} ref-stadium\_id \end{array}\to \begin{array}{c} stadium \end{array}$

The last step is to take all of this information and formulate it into a SPARQL query. The system has to decide which variables belong in the projection and if there are any additional elements in the query like aggregations, filters, set operations, group by or having clauses.

### 2.2 Ontologies

**Figure 2:**
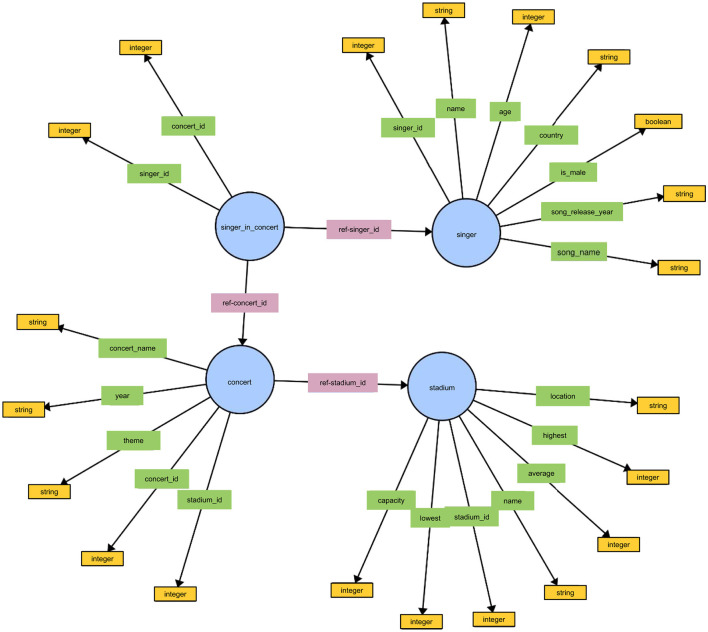
The figure depicts the ontology or T-box for the concert_singer knowledge graph in the Spider4SPARQL dataset. The figure shows the Classes in blue, the Object Properties in pink, the Datatype Properties in Green, and the DataTypes of each DataType Property in yellow.

One of the components that has largely been left out of building KGQA systems using LLMs, is the importance of ontologies. An ontology consists of classes, object properties, and datatype properties. An example of an ontology from the Spider4SPARQL dataset is shown in Figure 2. One of the key errors that KGQA systems make, is predicting triple patterns that do not exist or are in the wrong direction when connecting classes such as *stadium* and *concert* shown in Example 1. This challenge is specific to KGQA systems and does not apply to similar systems used for relational databases, such as text-to-SQL systems.

Let us explain this concept in more detail. For relational database management systems, the join condition of an inner join (other types of joins have not yet been implemented in text-to-SQL systems) between two tables can be declared in either direction. Consider the following two examples:

SELECT * FROM Table A join Table B on **A.id = B.id**

will produce the same results as:

SELECT * FROM Table A join Table B on **B.id = A.id**

The order or direction in which the join attributes (in bold above) are specified is irrelevant for SQL.

However, this is not the case for SPARQL. Here, the order or the direction of connecting classes (i.e., performing a join) is relevant since the ontology is a labeled *directed graph*. A connection between classes in one direction may not exist in another direction and will therefore return an empty result set.

The ontology in Figure 2 shows a connection going from the Class *concert* toward the Class *stadium*. A SPARQL query using the triple pattern shown in Example 2 would return an empty result set because this connection does not exist in the knowledge graph.

**Example 2:**

$\begin{array}{c} stadium \end{array}\to \begin{array}{c} ref-stadium\_id \end{array}\to \begin{array}{c} concert \end{array}$

The ontology contains the information that shows how classes are connected to one another, and is therefore crucial information when implementing any type of KGQA system. More evidence of this assertion is shown in the experiments and results in the next section.

## 3 Prompting frameworks

A variety of prompting strategies and frameworks have been introduced in order to take advantage of the expressive power of LLMs and their ability to understand natural language. These techniques attempt to exploit the ability of LLMs to acquire knowledge from vast amounts of text data, including code constructs, during the training process.

For the purpose of this analysis, we categorize the prompting frameworks into the following categories:

1. *Baseline prompt*: this category includes methods that leverage ontological information to enhance prompt effectiveness.
  (a) Our baseline prompt incorporates an ontology to provide contextual information to the LLM.
  (b) This category establishes a baseline performance for Knowledge Graph Question Answering with LLMs. This serves as a foundation for comparison with subsequent methods.
2. *Few-shot prompting*: this category explores the impact of incorporating few-shot examples into the baseline prompt.
  (a) We investigate the impact of supplementing the baseline prompt with few-shot examples, exploring both random and similarity-based examples.
3. *Query-optimized prompting*: methods in this category focus on modifying the structure or content of the query itself.
  (a) Explain-then-translate: this approach adds a chain-of-thought explanation to the SPARQL query, aiming to improve the LLM's understanding of the desired output.
  (b) Question decomposition meaning representation: this method breaks down the natural language question into smaller components, providing the LLM with a more structured input.
4. *Iterative prompt optimization*: this category evaluates a method that leverages the LLM's capabilities to refine a prompt.
  (a) Optimization by PROmpting:this framework explores the LLM's ability to self-improve the prompt through iterative feedback and refinement.

### 3.1 Baseline prompt with ontology

In order to determine the impact of target prompting techniques, we introduce a simple baseline prompt against which we compare and evaluate more complex methodologies. The baseline prompt includes a simple instruction about the task, information about the SPARQL prefixes, as well as the ontology. Example 1 shows the baseline prompt which is also later used in Section 4.3.1.1:

Example 1 **Baseline prompt. The “< >” placeholders denote specific variables.**


Given an input question create a syntactically
correct SPARQL query.
 
The prefix for the queries is PREFIX : <http://valuenet/ontop/>.
 
Use the following ontology: 
 
<Ontology> 
 
[Q]: <Natural Language Question>
[SPARQL]: <SPARQL Query>
[Q]: <Target Natural Language Question>
[SPARQL]:

### 3.2 Random few-shot prompting

In this method, we employ the baseline prompt with progressively increasing numbers of examples, pushing the limits allowed by the LLM's context window. This prompting method uses the same prompt and structure as the baseline method and is designed to interpret if simply giving the model more examples will produce better output.

### 3.3 Similarity-based few-shot prompting

In dynamic few-shot prompting (which we call *similarity-based*), an approach developed at Microsoft Research, the most relevant examples from the training data for each specific task are chosen based on their *similarity* to the current input (Nori et al., 2023). These similar questions are then used as the few-shot examples for prompting the model. Several open-source frameworks designed for building applications that use large language models, e.g., Langchain,^2^ offer functions that integrate dynamic few-shot prompting natively. This approach leverages the training data effectively without the need for extensive fine-tuning, which requires massive amounts of computational resources.

### 3.4 Explain-then-translate (ETT)

Explain-then-Translate (Tang et al., 2023) is a chain of thought type prompting method used to improve translation from one programming language to another. In this framework, an LLM generates a natural language explanation for the code, which is then used in the prompt. This techniques introduces three different types of explanations: explain (exp), explain line by line (lbl), and explain line by line in detail (lbl-d). This method was shown to consistently improve translation from Python into 18 target programming languages, especially in the zero-shot setting.

### 3.5 Question decomposition meaning representation (QDMR)

QDMR (Wolfson et al., 2020) is an approach to question understanding that decomposes complex natural language questions into simpler ones. It provides a language-agnostic way to represent the meaning of questions by breaking them down into smaller steps. Similar to query languages like SQL or SPARQL, QDMR uses a sequence of operations to select entities, retrieve information, and aggregate results. QDMR has been shown to improve question answering tasks and simplify complex logical forms used in semantic parsing. In contrast to previous frameworks, QDMR focuses on explaining natural language questions, while ETT focuses on explaining programming languages. We transform natural language questions into their QDMR using the method and model outlined in Wolfson et al. (2020).

### 3.6 Optimization by PROmpting (OPRO)

The OPRO approach to prompting from Google's Deep Mind was introduced early 2023 by Yang et al. (2024). OPRO proposes using LLMs as self-optimizers for their own prompts. The pipeline begins with a “meta-prompt” that feeds the LLM with past attempts (prompts with their performance scores, or human generated prompts with scores) and examples of the desired task (i.e., training data). The LLM then continuously suggests new prompts based on this information. These new prompts are evaluated and the best ones become part of the meta-prompt for the next iteration. This cycle allows OPRO to discover the most effective prompt for a specific task. 3.5% examples from the GSM8K dataset (Cobbe et al., 2021) were sampled and put through the OPRO pipeline. The results show that the OPRO framework outperforms the best manually developed prompts like “Let's think step by step” (Kojima et al., 2022) on the GSM8K dataset by 8.4%.

## 4 Experiments and results

This section details the experiments and results of translating natural language questions to SPARQL using the following six prompting methodologies: (1) Baseline, (2) Random Few-Shot Prompting, (3) Similarity-Based Few-Shot Prompting, (4) Explain-then-Translate (ETT), (5) Question Decomposition Meaning Representation (QDMR) and (6) Optimization by PROmpting (OPRO).

### 4.1 Benchmark dataset

For all our experiments we used the Spider4SPARQL benchmark (Kosten et al., 2023), which is currently considered as the most challenging benchmark for evaluating KGQA. This benchmark contains 9,693 natural language questions consisting of a training set of 8,659 NL/SPARQL-pairs and a test set of 1034 NL/SPARQL-pairs covering 166 multi-domain knowledge graphs and ontologies. The queries have a complexity of easy, medium, hard and extra-hard according to the Spider hardness definition (Yu et al., 2018).

### 4.2 Models and parameters

#### 4.2.1 OpenAI

For the experiments we used OpenAI's gpt-3.5-turbo-0125 model. The average processing time per run (inferencing 1,034 queries of the test set) was 30–40 min. For each experiment we performed 10 runs. We ran the queries using the OpenAI API. We set the temperature at 0 to ensure more deterministic results and set the top_p at 1. The total costs of these experiments was 163 USD.

#### 4.2.2 Code Llama

For comparison with the commercial model from OpenAI, we performed experiments with an open source model, Code Llama. Code Llama, from Meta, is built on Llama2 and has been specifically trained to handle a variety of programming tasks. It significantly outperforms other open source code-specific models (Roziére et al., 2024). For our experiments, we used and Code Llama—Instruct with 13b parameters.^3^

We set up and ran the model with the Ollama framework.^4^ During tests with the larger 34b and 70b Instruct models, the models frequently refused to produce SPARQL queries “due to safety concerns.”

These experiments were run on a gpu_1x_a100_sxm4 machine, rented from the Llambda GPU Cloud.^5^ The full GPU specs are provided in Table 1. The total costs for renting the GPU was 51.45 USD.

**Table 1:** The table shows the specs for the GPU used to process the queries with the Code Llama model.

**VRAM/GPU**	**vCPUs**	**RAM**	**STORAGE**	**PRICE**
40 GB	30	200 GiB	512 GiB SSD	$1.29/h

The processing times with the A100 GPU were similar to the processing times from the OpenAI API for the 1, 3, and 5 shot experiments. For the 10-shot experiments the processing time doubled.

### 4.3 Experiments

#### 4.3.1 Prompting with the ontology

We will now evaluate the performance of our six prompting methods using 10-fold cross-validation for the test set of 1,034 NL/SPARQL-pairs. This results in inferencing a total of 10,340 queries.

##### 4.3.1.1 Baseline

As a baseline experiment, we use the simple prompts introduced in Section 3.1. The execution accuracy of the baseline for translating to 1,034 natural language questions to SPARQL using zero-shot experiments is 8% for GPT-3.5 (see Table 2).

**Table 2:** Random few-shot prompting: the table shows the average execution accuracy of up to 30 shot experiments over 10 runs.

**Model**	**# of shots**	**Accuracy with ontology**
GPT-3.5	0 (Kosten et al., 2023)	8%
	1	46%(±1.16%)
	3	49%(±1.21%)
	**5**	**51**%(±0.70%)
	10 (Kosten et al., 2023)	45%(±4.41%)
	**20**	**50%** (±0.56%)
	30	49% (±1.03%)
Code Llama 13b	0	7%(±0%)
	1	7%(±0.57%)
	**3**	**16%**(±0.83%)
	**5**	**16%**(±0.76%)
	10	12%(0.79±%)

All shots are chosen randomly. We show the accuracy with the ontology used in the prompt. Bold values indicate the highest accuracy.

##### 4.3.1.2 Random few-shot prompting

To investigate the impact of training data size on model performance, we conduct experiments using randomly chosen natural language/SPARQL pairs. These shots were chosen from the training set of 8,659 NL/SPARQL-pairs. Both GPT-3.5 and Code Llama 13b have a context window of 16K. With GPT-3.5 we experimented with up to 30 examples. For Code Llama 13b we experimented with up to 10 examples. Running experiments with 20–30 examples for Code Llama 13b proved infeasible due to both cost constraints and processing times that exceeded the responsiveness real-world users expect.

Table 2 shows the average execution accuracy over 10 runs along with the errors in brackets. Note that the impact of using more than five training examples has not been previously explored in the literature. Interestingly, the results presented in Table 2 demonstrate that increasing the number of training examples to 30 decreases performance compared to 20 examples. However, the highest accuracy of 51% for GPT-3.5 and 16% for Code Llama, respectively, is achieved with only five training examples. These findings suggest that there may be a tipping point beyond which additional training data can hinder performance.

These initial experiments revealed a substantial performance gap between GPT-3.5 and Code Llama. GPT-3.5 outperformed Code Llama by a significant margin of 35%. To maximize the efficiency of our research and ensure the results are most relevant to our specific task, we opted to focus our efforts on the higher-performing model.

##### 4.3.1.3 Similarity-based few-shot prompting

This prompting methodology does not include randomly chosen shots but rather shots that are selected based on their semantic similarity to the target natural language question. We use the all-MiniLM-L6-v2 sentence encoder from HuggingFace^6^ to encode the natural language questions and cosine similarity to measure the distance between the target natural language question and the examples from the train set. The training samples are only chosen based on the natural language question. The results for 1, 3, and 5-shot experiments of GPT-3.5 are shown in Table 3.

**Table 3:** Similarity-based few-shot prompting: the table shows the execution accuracy of the 1, 3, and 5 shot experiments for GPT-3.5.

**# of shots**	**Accuracy with ontology**
1	39%
3	42%
5	**46**%

Bold values indicate the highest accuracy.

We limit these experiments to the 1, 3, and 5-shot setting to maintain a high degree of semantic similarity between the examples and the target question. While it would be technically possible to use more shots, this could introduce irrelevant information that could hinder the model's ability to learn the concepts relevant to the target question.

Table 3 shows that similarity-based few shot prompting performs 4% worse than random few-shot prompting shown previously in Table 2. There are several potential explanations for this drop in accuracy. One possible explanation is overfitting in similarity-based example selection. By choosing examples that are too closely aligned with the target question, the model might struggle to generalize to slightly different query structures or variations in natural language expressions. Randomly selecting examples, on the other hand, might introduce a degree of diversity that exposes the model to a broader range of query patterns and SPARQL constructs. This exposure could enable the model to develop a more robust understanding of the task, leading to better generalization. Further research is warranted to expose the underlying reasons for this phenomenon. Future work could explore the optimal number of examples for few-shot LLM-based KGQA or investigate the effectiveness of combining random and similarity-based example selection strategies to gain a more comprehensive understanding of this observation.

##### 4.3.1.4 Explain-then-Translate

For evaluating the Explain-then-Translate prompt engineering framework, we sample 30% of the Spider4SPARQL training set (2,598 queries), including only hard and extra hard questions, as previous few-shot experiments show produces the highest accuracy (Kosten et al., 2023). We provide the NL/SPARQL-pairs to GPT-3.5 and ask it to generate explanations.

Example 2 shown below is an extra hard query from a knowledge graph about academia. The example shows the basic *exp* explanations prompt, the NL/SPARQL-pair and the explanation. Further examples of *line by line* and *line by line in detail* explanation prompts and their generated responses are included in the Example 10 in the Appendix.

Example 2 **Explanation Prompt.**


You are a helpful AI assistant who understands the SPARQL Protocol and
RDF Query Language. Can you explain what this SPARQL script does with a
couple of sentences? The goal of the explanation is so that a reader can
easily rewrite the question in SPARQL.
  
[Q]:
Return me the keyword, which has been contained by the most number
of papers in VLDB conference.
  
[SPARQL]:
SELECT ?T1_keyword (count(*) as ?aggregation_all) WHERE {
?T1 a :keyword.
?T14 a :publication_keyword.
?T14 :publication_keyword#ref-kid ?T1.
?T1 :keyword#keyword ?T1_keyword.
?T2 a :publication.
?T2 :publication#title ?T2_title.
?T14 :publication_keyword#ref-pid ?T2.
?T3 a :conference.
?T3 :conference#name ?T3_name.
?T2 :publication#ref-cid ?T3.
FILTER(?T3_name = 'VLDB').}
GROUP BY ?T1_keyword
ORDER BY DESC ((count(?T2_title) as ?aggregation)) LIMIT 1
  
[Explanation]:
This SPARQL script retrieves the keyword that appears in the most number
of papers in the VLDB conference. It does this by querying for papers that
have a keyword, publications that have a title, conferences that have a
name, and linking them together using various properties. It then filters
the results to only include papers from the VLDB conference and groups the
results by keyword. Finally, it orders the results in descending order bas-
ed on the count of titles and limits the output to only the keyword with
the highest count.

Let us analyze the results for GPT-3.5. We performed 1, 3, and 5-shot experiments with the setting *exp*. The shots for each prompt were randomly selected.

Experiments using 3 and 5-shots with the *lbl-d* (line-by-line in detail) explanation setting with the ontology were not feasible because they exceeded the context window of 16,385 tokens.

Table 4 shows the results of the experiments for the Explain-then-Translate framework. Increasing the number of shots included in the prompt from 1 to 5, led to a 3% increase in accuracy.

**Table 4:** Explain-then-translate: the table shows the execution accuracy of 1, 3, and 5 shot experiments for GPT-3.5 in the *exp* setting.

**# of shots**	**Accuracy with ontology**
1	47% (±0.80%)
3	49% (±1.03%)
**5**	**50%** (±0.80%)

Bold values indicate the highest accuracy.

##### 4.3.1.5 Question decomposition meaning representation

For evaluating the QDMR framework, we utilize the same hard and extra hard training examples (30% of the Spider4SPARQL training set, i.e., 2,598 queries) as in the Explain-then-Translate experiments.

Example 3 shows the input that the model receives in the 1-shot experiments, i.e., the ontology (abridged version shown below), an example from the training data with a natural language question, a SPARQL query, a QDMR explanation and the target question.

Example 3 **QDMR Explanation Prompt.**


Given an input question and an ontology create a syntactically correct
SPARQL query.
The prefix for the queries is PREFIX : <http://valuenet/ontop/>.
Use the following ontology:
‘kg_id': ‘concert_singer',
‘classes': [‘concert', ‘singer', ‘singer_in_concert', ‘stadium'],
‘object_properties': [‘concert#ref-stadium_id'],
‘data_properties': [‘concert#concert_id', ‘concert#concert_name'],
  
Please use the following examples to better understand this task:
[Q]: who does Noah A Smith work with ?
[SPARQL]:
SELECT DISTINCT ?T1_authorid WHERE {
?T1 a :author.
?T1 :author#authorid ?T1_authorid.
?T1 :author#authorname ?T1_authorname.
FILTER(?T1_authorname = 'Noah A Smith').}
  
[Explanation]:
‘return noah a smith ',
‘return who does #1 work with'
  
[Q]:How many singers do we have?
[SPARQL]:

The best QDMR experiment including the ontology has an accuracy of 49% (see Table 5).

**Table 5:** Question decomposition meaning representation: the table shows the execution accuracy of 1, 3, and 5 shot experiments using GPT-3.5.

**# of shots**	**Accuracy with ontology**
1	46% (±1.30)
3	48% (±0.77)
5	**49% (±0.65)**

Bold values indicate the highest accuracy.

Table 5 shows that QDMR performs 1% below Explain-then-Translate (ETT, previously shown in Table 4) across the different numbers of shots. The two different frameworks focus on different aspects of explanations. ETT was designed as a prompt engineering strategy focused on explaining programming languages, while QDMR centers on explaining natural language questions. This difference in focus could contribute to their performance discrepancies in the context of LLM-based KGQA. ETT may have an advantage because the step-by-step explanations of the SPARQL queries might provide more direct guidance for the LLM to map natural language elements to SPARQL constructs. QDMR's emphasis on decomposing natural language questions into simpler units might not be as effective in directly addressing the challenges of SPARQL generation. While breaking down the natural language question can help with understanding, it might not offer the same level of explicit mapping to SPARQL syntax and structure as ETT. To further investigate the performance disparity between ETT and QDMR in the context of SPARQL-based KGQA, future research could explore the impact of explanation granularity for SPARQL query generation. Such analysis would reveal the optimal level of granularity that balances natural language understanding with effective SPARQL query generation.

##### 4.3.1.6 Optimization by PROmpting

We perform experiments using the OPRO framework. In the first phase, the model is given an optimization prompt as shown in the example below, and produces optimized queries. These optimized queries are then evaluated against the entire Spider4SPARQL training data set of 8,659 queries. In this phase, the model is given the optimized prompt, the ontology and a natural language question. The prompt that performs best over the training data set is then used for evaluating the test set.

The best prompts generated by this framework are able to answer 11% of the questions in the zero-shot scenario on the test set. This is a 3% improvement over the performance of the manually generated prompt from the baseline experiments of the Spider4SPARQL benchmark (Kosten et al., 2023)—see also Table 2.

Let us now analyze the optimization prompt (see Example 4), the generated prompts (see Example 5), and the generation prompt (see Example 6) employing the OPRO framework.

Example 4 **Optimization Prompt.**


I have some texts along with their corresponding scores.
The texts are arranged in ascending order based on their scores, where
higher scores indicate better quality.
Write your new text that is similar but different from the old ones.
Write the text in square brackets.

Example 5 **Generated Prompts.**


1- Let's approach this systematically. Provide the SPARQL query only. No additional information needed.
   score: 70
2- text: Let's approach the task systematically. Provide only the SPARQL query. No additional information.
3- text: Let's carefully analyze the data. Only respond with the SPARQL query. Nothing more.
   score: 70
4- text: Let's tackle the challenge with precision. Respond with the SPARQL query only. No additional information needed.
   score: 70
5-text: Let's approach the task systematically. Provide a concise SPARQL query as your response. No additional information needed.
6- text: Let's approach this systematically. Provide the SPARQL query only. No additional information.
7- text: Let's approach this systematically. Provide the SPARQL query only, without any additional information.
8- Write the SPARQL. Only answer with the SPARQL. Nothing else.

Example 6 **Generation Prompt. Without being prompted to do so, the model generated its own scores e.g. “score: 70” for 3 of the prompts.**


PREFIX : <http://valuenet/ontop/>
Given the ontology:
{‘kg_id': ‘concert_singer',
‘classes': [‘concert', ‘singer', ‘singer_in_concert', ‘stadium'],
‘object_properties': [‘concert#ref-stadium_id',
‘singer_in_concert#ref-concert_id', ‘singer_in_concert#ref-singer_id'],
‘data_properties': [‘concert#concert_id', ‘concert#concert_name',
‘concert#stadium_id', ‘concert#theme', ‘concert#year', ‘singer#age',
‘singer#country', ‘singer#is_male', ‘singer#name', ‘singer#singer_id',
‘singer#song_name', ‘singer#song_release_year',
‘singer_in_concert#concert_id',‘singer_in_concert#singer_id',
‘stadium#average',‘stadium#capacity', ‘stadium#highest', ‘stadium#location',
‘stadium#lowest', ‘stadium#name', ‘stadium#stadium_id']}
[Q]:
How many singers do we have?
[text]:
Let's approach this systematically. Provide the SPARQL query only.
No additional information needed.
score: 70

The optimization prompt shown in Example 4, informs the model about the task it will be performing, i.e. writing new prompts that should have higher scores than the old prompts. The model was set to output 10 optimized prompts, for brevity we have included the de-duplicated list of eight generated prompts in Example 5. The scores shown in some of the generated prompts are hallucinated by the model and have no relation to the actual performance of the prompts.

The following are examples of the types of queries that were generated by GPT-3.5 using the prompts generated in the OPRO framework.

The first prompts in the optimization process produced queries like Example 7. It seems like the model has some knowledge of the SPARQL query language because it is able to produce output that resembles triples. However, this output is syntactically incorrect, due to the added **it]** at the end of the query.

Example 7 **Using the prompt generated by the OPRO framework (first iteration).**


Question: List the dates of debates with number of audience bigger than 150.
  
Query: SELECT ?date WHERE {
?debate debate#date ?date;
debate#num_of_audience ?audience.
FILTER (?audience > 150) }it]

In Example 8 the model unsuccessfully attempts to format the query in a URL-like format, even though it has been provided with the necessary and ontology.

Example 8 **Using the prompt generated by the OPRO framework (second iteration).**


Question: What is the highest salary among each team? List the team name, id and maximum salary.
  
Query: SELECT ?team_name ?team_id (MAX(?salary) AS ?max_salary)
WHERE { ?team a <team\#team>;
<team#name> ?team_name;
<team#team_id> ?team_id.
?salary a <salary#salary>;
<salary#team_id> ?team_id;
<salary#salary> ?salary_value. }
GROUP BY ?team_name ?team_id

Over the course of the iterations of self-improving prompts, the model did manage to produce queries that were syntactically correct. The query in Example 9 would be executable, except that the model added its own prefix, which does not match the IRI's in the underlying graph. Removing the incorrect prefix could be done in a post-processing step.

Example 9 **Using the prompt generated by the OPRO framework (third iteration).**


Question: What is the id and first name of all the drivers who participated in the Australian Grand Prix and the Chinese Grand Prix?
  
Query: PREFIX : <http://example.org/formula_1/>
SELECT ?driverid ?forename WHERE {
?race1 a :races;
:name ‘‘Australian Grand Prix~;
:raceid ?raceid1.
?race2 a :races;
:name ‘‘Chinese Grand Prix~;
:raceid ?raceid2.
?result1 a :results;
:raceid ?raceid1;
:driverid ?driverid.
?result2 a :results;
:raceid ?raceid2;
:driverid ?driverid.
?driver a :drivers;
:driverid ?driverid;
:forename ?forename. }

The OPRO framework demonstrates that models can improve the prompts they are given, leading to better 3% results for 5 shots with respect to 1 shot (see Table 6). One area for future research would be to explore the OPRO framework in combination with the top-performing Query-Optimized-Prompting Framework, *ETT*.

**Table 6:** Optimization by prompting framework: the table shows the execution accuracy of the 1, 3, and 5 shot experiments for GPT-3.5.

**# of shots**	**Accuracy with ontology**
1	44% (±1.11)
3	**49%** (±1.077)
**5**	**49%** (±1.19)

Bold values indicate the highest accuracy.

#### 4.3.2 Ablation study: prompting without the ontology

Finally, we conduct experiments across three different prompting frameworks without using the ontology of the knowledge graph. The prompting frameworks we use are (1) Baseline, (2) ETT (both the explain and explain line by line explanations), and (3) QDMR.

Table 7 shows that not including the ontology leads to poor results. We evaluated the impact of ontologies on prompting effectiveness across several frameworks. Our analysis revealed a difference of 26% in accuracy between frameworks that used ontologies and those that did not. The best-performing framework without an ontology achieved an accuracy of 13%, while the least accurate framework with an ontology reached 39%. Due to this performance gap, we focused further investigation on frameworks that employed ontologies. Therefore, we did not conduct additional experiments with the remaining frameworks in the *without ontology* setting.

**Table 7:** The table shows the execution accuracy for prompts that do not include the ontology.

**Model**	**# of shots**	**Accuracy without ontology**
Baseline	1	8%
	3	10%
	5	10%
ETT-exp	1	8%
	3	13%
	5	9%
ETT-lbl	1	7%
	3	13%
	5	9%
QDMR	1	11%
	3	13%
	5	13%

The full length description for the abbreviations in the ETT framework are as follows: explain (exp), explain line by line (lbl).

## 5 Error analysis

In the following section, we analyze the queries that were systematically incorrectly inferred (representing 34% of the test set) across the three top performing frameworks using the knowledge graph ontology, i.e., frameworks that achieved 50%–51% accuracy.

**Figure 3:**
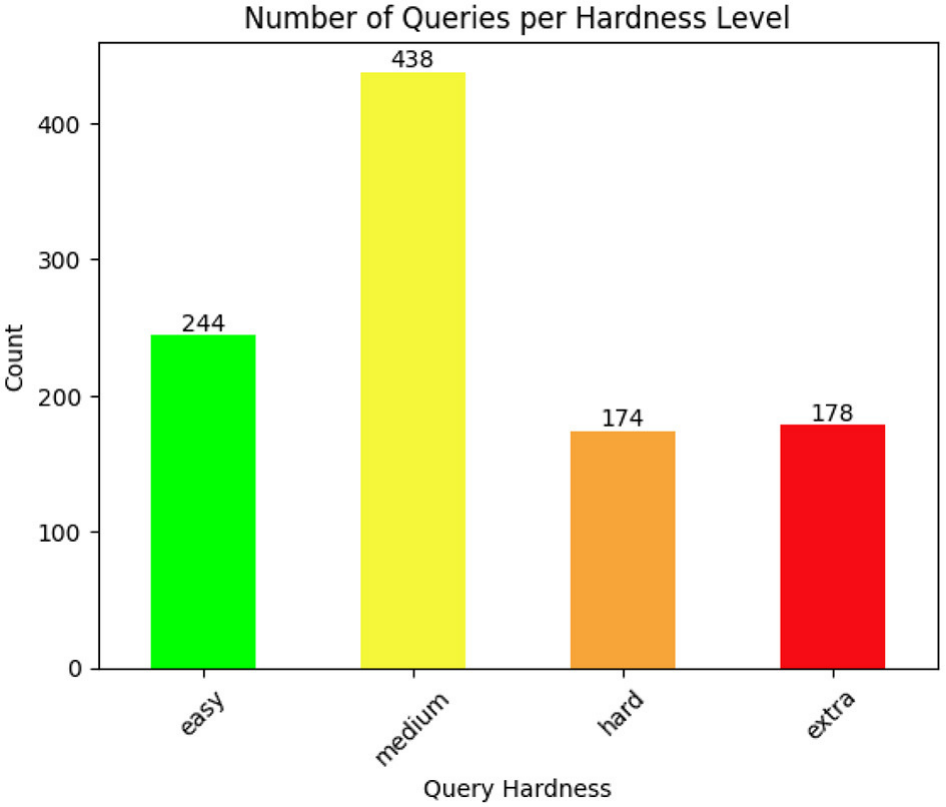
The figure shows the number of queries per hardness level according to the Spider Hardness Framework.

We evaluate the queries using the Spider hardness *easy, medium, hard* and *extra hard* as previously introduced. The number of queries per hardness level are shown in Figure 3.

In the following analysis, we consider three different types of output:

1. Match: queries that produce results that match the ground truth.
2. No match: queries that produce results but do not match the ground truth.
3. Not executable: queries that are not able to be executed against the RDF triple store.

**Figure 4:**
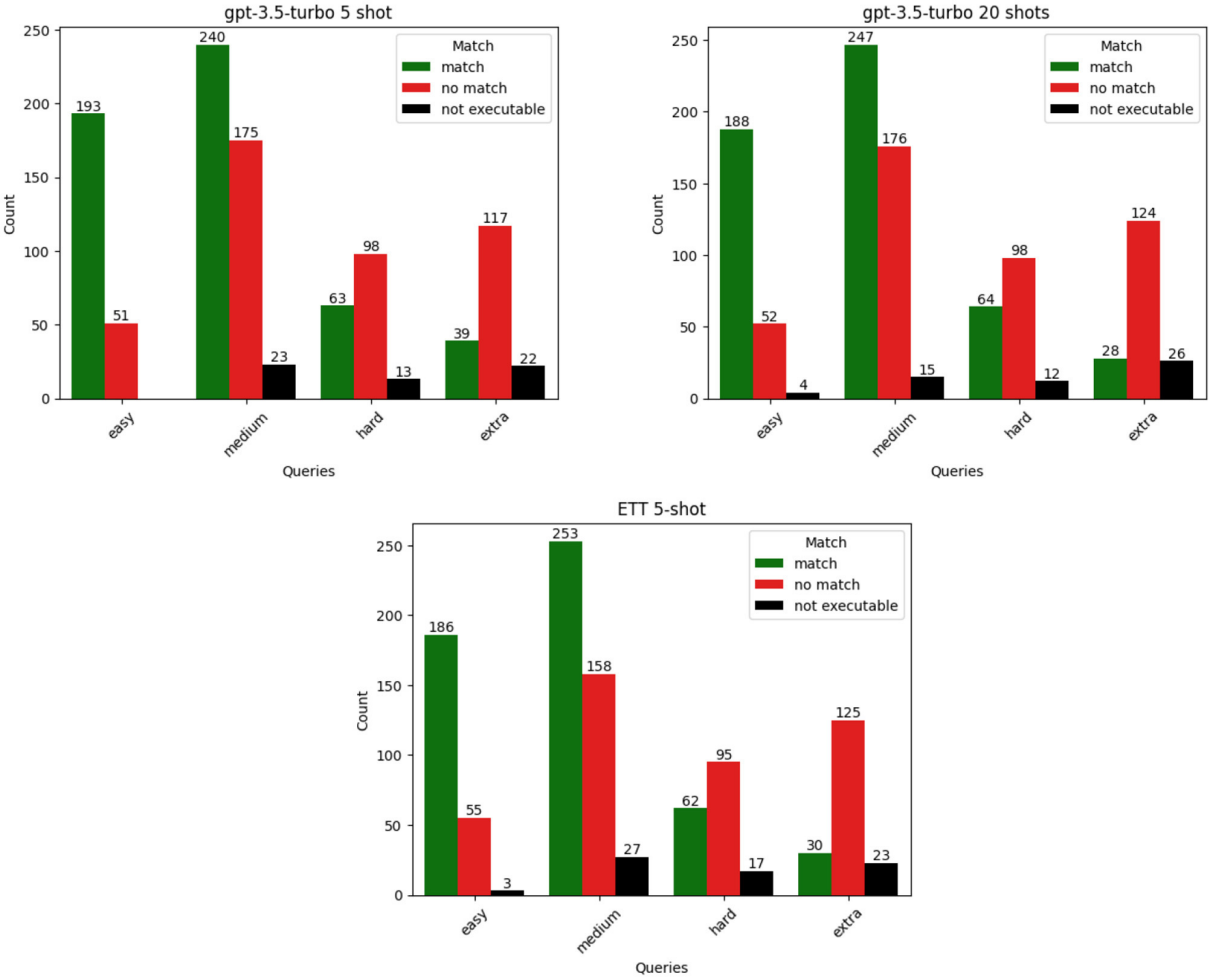
Execution results per query hardness for three top performing frameworks: random few-shot prompting with 5-shots and 20-shots, and ETT 5-shot.

The Figure 4 shows that models are more successful at translating the easy and medium type queries from natural language into SPARQL across the prompting frameworks. We can also see that with hard and extra hard type queries the models struggle to produce accurate answers.

### 5.1 Types of errors

Let us analyze the types of errors that occur when translating natural language to SPARQL in more detail. All the examples we address here are shown in the Appendix.

#### 5.1.1 Syntax errors

3% of the queries could not be executed due to syntax errors. Some of the syntax errors were due to the model producing an explanation for the generated query, even when prompted to “only produce a SPARQL query”. This kind of error could easily be resolved in a post-processing step.

Example 11 in the Appendix, demonstrates a different kind of syntax error in the **Filter** of the query. Instead of using a comparison operator against a variable, the model has tried to use an incomplete triple pattern **?T1 :pets#pet_age** in the filter. The query is also missing the triple required to access the target variable **?T1_pet_age** for the filter.

#### 5.1.2 Missing triples

Example 12 in the Appendix shows the ground truth query and the generated query, which answers the question, “Return the names of all the poker players.” The model has been provided with the ontology in the prompt, so the information about connections between classes is available. In this case, the model has failed to use the information provided in the ontology, which only has two classes **people** and **poker_player**.

Example 13 in the Appendix answers the question, “What are the countries having at least one car maker? List name and id.” The generated SPARQL query has a few errors, most notably it is missing a HAVING-clause even though this keyword is contained in the natural language question. Additionally, this query is missing the class **countries**, which contains the property **countryname**. The model has not connected the **countries** class with the reference in the natural language question. It has also not made use of the information in the ontology that shows that the classes **car_makers** and **countries** are connected.

#### 5.1.3 Incorrect variable chosen for aggregation in projection

For this type of error, the model chooses the wrong variable for an aggregation. Example 14 in the Appendix is syntactically correct, however, since the variable **?T1_petid** does not exist in any of the triples in the query, the query returns an empty set.

The natural language question for Example 15 in the Appendix is “how many cars were produced in 1980?”. In this example, not only has the model forgotten to include a triple with the variable from the projection **?T1_id**, but it misinterpreted the query which should have simply counted all of the rows that met the criteria set by the filter i.e., Select (count (*) as ?agg).

#### 5.1.4 Limitations in filter generation

The following examples demonstrate challenges that KGQA systems face in answering questions that require a filter. Example 16 in the Appendix answers the question “What are the names of the singers who are not French citizens?” The literal that corresponds to **singer#citizenship** is the country name **France** not the adjective **French**. In this case, the model simply used the named entity from the natural language question. This kind of error could be solved with a pre-processing pipeline that informed the model about literals in the graph that are semantically similar to the named entities in the natural language questions.

Example 17 in the Appendix is from a knowledge graph about TV shows and answers the question, “When did the episode ‘A Love of a Lifetime' air?”. In this case, the model has included the triple needed for the filter **?T1_episode**, but chose the wrong variable, i.e., **?T1_series_name**. It would appear in this instance that the model has misunderstood the difference between an episode of a TV show and a series.

## 6 Conclusion

In this paper we presented experiments and a thorough evaluation of six different prompting frameworks, namely (1) Baseline, (2) Random Few-Shot Prompting, (3) Similarity-Based Few-Shot Prompting, (4) Explain-then-Translate (ETT), (5) Question Decomposition Meaning Representation (QDMR), and (6) Optimization by PROmpting (OPRO). The recent advancements in prompt engineering frameworks within the research community are aimed at improving few-shot LLM capabilities in tasks like code translation and question answering. To the best of our knowledge, we are the first to perform rigorous experiments with few shot selection by performing 10-fold experiments for translating natural language questions into SPARQL queries.

The top performing framework is *random few-shot prompting* with no additional explanations for the natural language questions or SPARQL queries. Our experiments also indicate that the more advanced prompting engineering frameworks such as ETT, QDMR or OPRO do not yield significantly better results. The 5-shot random few-shot prompting experiment outperformed the 20-shot random few shot prompting experiment by 1% indicating that more shots does not necessarily help the model to produce accurate queries. Finally, a top score of 51% indicates that complex Knowledge Graph Question answering is still an unsolved research question, even for commercial large language models.

## Data Availability

Publicly available datasets were analyzed in this study. This data can be found at: https://github.com/ckosten/Spider4SPARQL.

## Footnotes

^1^The following link was accessed on February, 21st 2024 and shows current statistics about ChatGPT usage https://www.demandsage.com/chatgpt-statistics.

^2^ https://api.python.langchain.com/en/latest/example_selectors/langchain_core.example_selectors.semantic_similarity.SemanticSimilarityExampleSelector.html

^3^ https://ollama.com/library/codellama:13b

^4^ https://ollama.com/

^5^ https://cloud.lambdalabs.com

^6^ https://huggingface.co/sentence-transformers/all-MiniLM-L6-v2
