# Supplementary material for: Evaluating the effectiveness of prompt engineering for knowledge graph question answering
Source: Front Artif Intell. 2025 Jan 13;7:1454258. doi: 10.3389/frai.2024.1454258 (PMC11770024; doi:10.3389/frai.2024.1454258)
Supplement: Supplementary file 1 [file Data_Sheet_1.pdf]

Supplementary Material

1 APPENDIX

```
Question:How many airlines are from USA?

Ground Truth: SELECT (COUNT(*) as ?aggregation_all) WHERE {
?t1 a :airlines .
?t1 :airlines#country ?t1_country .
FILTER(?t1_country = 'USA') .}

Generated SPARQL Query: SELECT (COUNT(?T1) as ?aggregation_all) WHERE {
?T1 a :airlines .
?T1 :airlines\#country ?T1_country .
FILTER(?T1_country = 'USA') .
}
[Explanation]:
This SPARQL script retrieves the count of airlines that are
from the USA. It does this by querying for instances of the "airlines" class
and filtering the results based on the country property. The count of the
   results is then calculated.
```

Example 1. Explanation Prompt included in the answer

```
Question: Find the id and weight of all pets whose age is older than 1.

Ground Truth: SELECT ?t1_petid ?t1_weight WHERE {
?t1 a :pets .
?t1 :pets#petid ?t1_petid .
?t1 :pets#weight ?t1_weight .
?t1 :pets#pet_age ?t1_pet_age .
FILTER(?t1_pet_age > 1) }

Generated SPARQL Query: SELECT ?T1_petid ?T1_weight WHERE {
?T1 a :pets .
?T1 :petspetid ?T1_petid .
?T1 :pets#weight ?T1_weight .
FILTER(?T1 :pets#pet_age > 1) }
```

Example 2. Syntax Error in Filter

```
Question: What are the names of poker players?

Ground Truth: SELECT ?t1_name WHERE {
?t1 a :people .
?t2 a :poker_player .
```

```
?t2 :poker_player#ref-people_id ?t1 .
?t1 :people#name ?t1_name .
?t1 :people#people_id ?t1_people_id .}

Generated SPARQL Query: SELECT ?T1_name WHERE {
?T1 a :poker_player .
?T1 :people#name ?T1_name . }
```

Example 3. Missing Triples #1

```
Question: What are the countries having at least one car maker? List name
   and id.

Ground Truth: SELECT ?t1_countryname ?t1_countryid WHERE {
?t1 a :countries .
?t2 a :car_makers .
?t2 :car_makers#ref-country ?t1 .
?t1 :countries#countryname ?t1_countryname .
?t1 :countries#countryid ?t1_countryid .
}
group by ?t1_countryname ?t1_countryid
having(count( *) >= 1)

Generated SPARQL Query: SELECT ?T1_countryname ?T1_countryid WHERE {
?T1 a :car_makers .
?T1 :car_makers#country ?T1_countryname .
?T1 :car_makers#id ?T1_countryid . }
```

Example 4. Missing Triples #2

```
Question: How many pets have a greater weight than 10?

Ground Truth: SELECT (COUNT(*) as ?aggregation_all) where {
?t1 a :pets .
?t1 :pets#weight ?t1_weight .
FILTER(?t1_weight > 10) .}

Generated SPARQL Query: SELECT (count( ?T1_petid) as ?
   aggregation_T1_petid_52 ) WHERE {
?T1 a :pets .
?T1 :pets#weight ?T1_weight .
FILTER(?T1_weight > 10) . }
```

Example 5. Incorrect Variable Chosen for Aggregation in Projection #1

```
Question: How many cars were produced in 1980?

Ground Truth: SELECT (COUNT(*) as ?aggregation_all) WHERE {
```

```
?t1 a :cars_data .
?t1 :cars_data#year ?t1_year .
FILTER(?t1_year = 1980)}

Generated SPARQL Query: SELECT (count( ?T1_id) as ?agg ) WHERE {
?T1 a :cars_data .
?T1 :cars_data#year ?T1_year .
FILTER(?T1_year = 1980) . }
```

Example 6. Incorrect Variable Chosen for Aggregation in Projection #2

```
Question: What are the names of the singers who are not French citizens?

Ground Truth: SELECT ?t1_name WHERE {
?t1 a :singer .
?t1 :singer#name ?t1_name .
?t1 :singer#citizenship ?t1_citizenship .
FILTER(?t1_citizenship != 'France') }

Generated SPARQL Query: SELECT ?T1_name WHERE {
?T1 a :singer .
?T1 :singer#name ?T1_name .
?T1 :singer#citizenship ?T1_citizenship .
FILTER(?T1_citizenship != 'French') .
```

Example 7. Limitations in Filter Generation #1

```
Question: What is the air date of TV series with Episode 'A Love of a
   Lifetime'?

Ground Truth: SELECT ?t1_air_date WHERE {
?t1 a :tv_series .
?t1 :tv_series#air_date ?t1_air_date .
?t1 :tv_series#episode ?t1_episode .
FILTER(?t1_episode = 'A Love of a Lifetime')}

Generated SPARQL Query:SELECT ?T1_air_date WHERE {
?T1 a :tv_series .
?T1 :tv_series#episode ?T1_episode .
?T1 :tv_series#air_date ?T1_air_date .
?T1 :tv_series#id ?T1_id .
?T1 :tv_series#series_name ?T1_series_name .
FILTER(?T1_series_name = 'A Love of a Lifetime') . }
```

Example 8. Limitations in Filter Generation #2
